# Recovery from Spatial Neglect with Intra- and Transhemispheric Functional Connectivity Changes in Vestibular and Visual Cortex Areas—A Case Study

**DOI:** 10.3389/fneur.2018.00112

**Published:** 2018-03-02

**Authors:** Julian Conrad, Rainer Boegle, Matthias Ertl, Thomas Brandt, Marianne Dieterich

**Affiliations:** ^1^Department of Neurology, Ludwig-Maximilians-Universität, Munich, Germany; ^2^German Center for Vertigo and Balance Disorders – IFB^LMU^ (DSGZ), Ludwig-Maximilians-Universität, Munich, Germany; ^3^Graduate School for Systemic Neuroscience (GSN), Ludwig-Maximilians-Universität, Munich, Germany; ^4^Clinical Neuroscience, Ludwig-Maximilians-Universität, Munich, Germany; ^5^Munich Cluster for Systems Neurology (SyNergy), Munich, Germany

**Keywords:** neglect, vestibular, spatial, visual, functional connectivity, caloric, parietal operculum 2

## Abstract

**Objective:**

Vestibular signals are involved in higher cortical functions like spatial orientation and its disorders. Vestibular dysfunction contributes, for example, to spatial neglect which can be transiently improved by caloric stimulation. The exact roles and mechanisms of the vestibular and visual systems for the recovery of neglect are not yet known.

**Methods:**

Resting-state functional connectivity (fc) magnetic resonance imaging was recorded in a patient with hemispatial neglect during the acute phase and after recovery 6 months later following a right middle cerebral artery infarction before and after caloric vestibular stimulation. Seeds in the vestibular [parietal operculum (OP2)], the parietal [posterior parietal cortex (PPC); 7A, hIP3], and the visual cortex (VC) were used for the analysis.

**Results:**

During the acute stage after caloric stimulation the fc of the right OP2 to the left OP2, the anterior cingulum, and the para/hippocampus was increased bilaterally (i.e., the vestibular network), while the interhemispheric fc was reduced between homologous regions in the VC. After 6 months, similar fc increases in the vestibular network were found without stimulation. In addition, fc increases of the OP2 to the PPC and the VC were seen; interhemispherically this was true for both PPCs and for the right PPC to both VCs.

**Conclusion:**

Improvement of neglect after caloric stimulation in the acute phase was associated with increased fc of vestibular cortex areas in both hemispheres to the para-hippocampus and the dorsal anterior cingulum, but simultaneously with reduced interhemispheric VC connectivity. This disclosed a, to some extent, similar but also distinct short-term mechanism (vestibular stimulation) of an improvement of spatial orientation compared to the long-term recovery of neglect.

## Introduction

The vestibular system subserves eye, head, and body coordination in space, postural control, and the perception of verticality and self-motion (1). Its involvement in “higher” cortical functions such as spatial memory, orientation, and navigation, as well as cognition is well recognized (2). In contrast to other sensory modalities, cortical vestibular areas are multisensory; they closely interact with visual and somatosensory input.

Spatial neglect is a heterogeneous disorder of spatial and non-spatial attention which leads to reduced awareness of stimuli in the contralesional hemispace (3, 4). It can affect the representation of one’s own body, the surroundings, or abstract space, such as imagined space, time lines, and number space (3). Imaging evidence suggests that spatial neglect should be regarded as a network disorder of spatial and non-spatial attentional systems (5, 6).

Typically a spatial hemineglect is caused by an ischemic stroke within the middle cerebral artery (MCA) territory. The lesioned areas in neglect patients are generally located in the perisylvian regions and the superior and middle temporal gyrus particularly within the right hemisphere, the inferior parietal lobule (IPL) and less frequently the inferior prefrontal cortex (4).

Several findings support the view that the vestibular system contributes to the manifestation of the neglect syndrome. For example, the causative lesions overlap with the vestibular cortical network, which characteristically shows a strong preponderance of the right hemisphere in right-handers (5, 7, 8). Further, vestibular stimulation can temporarily improve neglect symptoms; this was first demonstrated with caloric irrigation by Rubens (9). Thus, the integrity of cortical vestibular function seems to be important for maintaining maps for spatial orientation (10–12).

There is growing evidence that spatial orientation relies on vestibular signals (11, 13, 14), but their exact contribution as to the particular cortical structures and interconnections to other sensory cortical areas has yet to be demonstrated. It is unclear which areas of the vestibular cortical network are involved in the acute stage and how the spontaneous recovery is achieved within the multisensory attentional system.

The observation that vestibular stimulation, e.g., by calories, can transiently improve the disrupted awareness of visual stimuli in the contralateral neglected hemifield raises the question as to whether the spontaneous recovery over months is mainly based on a functional reorganization of the multisensory attentional network rather than a structural and functional restoration of the ischemic neuronal assemblies.

The aim of the current study was to evaluate, first, the acute beneficial effect of vestibular stimulation on neglect and, second, the spontaneous recovery from neglect within days to months. Methodologically we measured changes in resting-state functional connectivity (fc) of the core regions of the central vestibular and visual networks within the affected hemisphere and between the two hemispheres during the acute and chronic stages of the condition. The following questions were addressed in a patient with a typical spatial hemineglect during the acute stage compared to 6 months later when the patient had completely recovered: 1. is the improvement of spatial hemineglect after caloric stimulation in the acute stage associated with changes in fc within the cortical vestibular network, and if so, which areas are involved? 2. Does caloric stimulation lead to changes in cortical network interactions between vestibular, attentional, and/or visual networks? 3. Are these changes in the acute stage after vestibular stimulation similar to or different from the changes seen in fc after recovery of the initial deficit 6 months later? It is hypothesized that the changes in fc elicited by caloric vestibular stimulation in the acute stage involve connectivity patterns that later provide the basis for a robust amelioration in the chronic stage.

## Case Report and Methods

**Figure 1:**
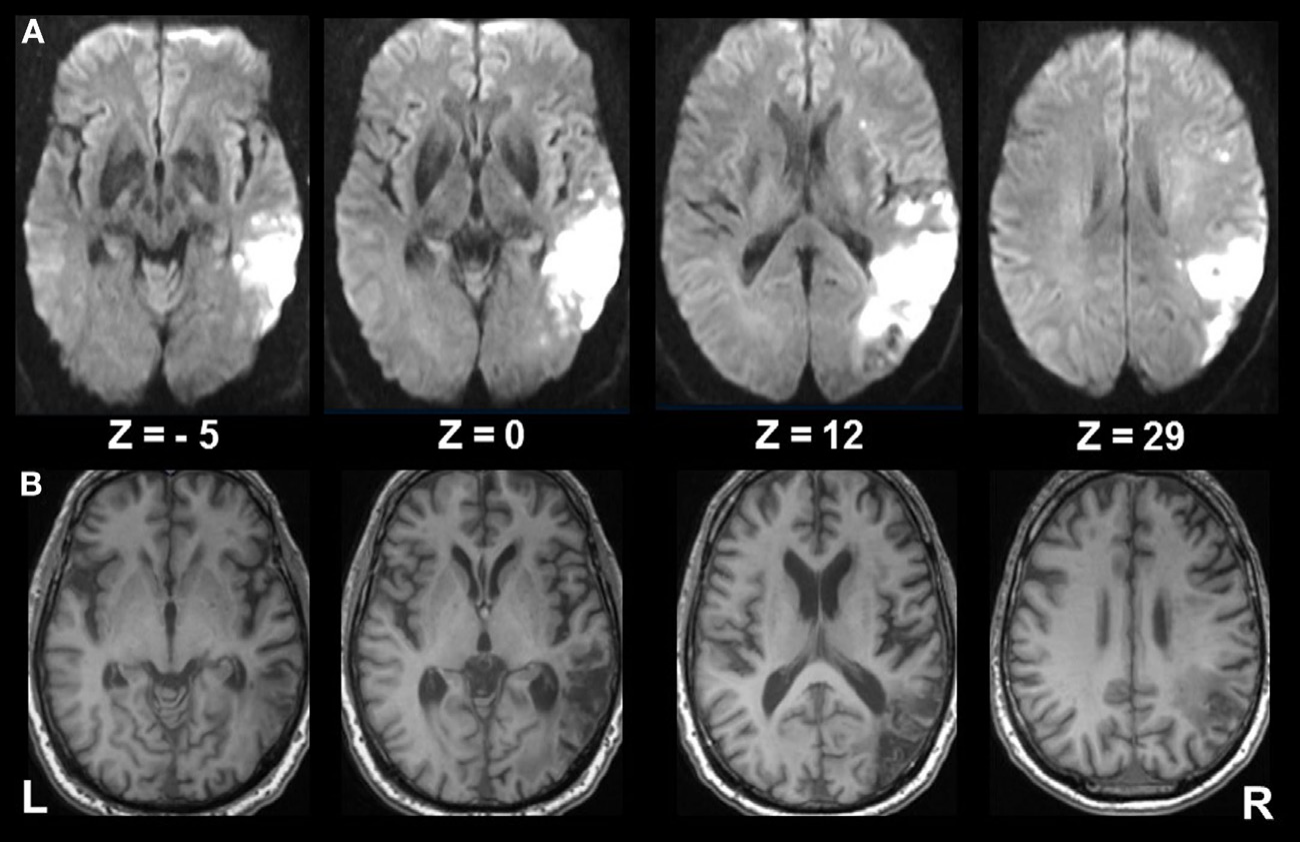
Extent of infarcted tissue in the acute and chronic phase. **(A)** Diffusion weighted imaging in the acute phase and **(B)** T1 FSPGR at 6 months show the extent of infarcted tissue in the right middle cerebral artery territory involving mainly the inferior parietal lobule (ANG; bordering the intraparietal sulcus), V5, and the temporo-occipital junction, sparing the posterior insula, parietal opercular cortex, and posterior parietal cortex. Subcortical structures involve the superior longitudinal fasiculus (II, III), inferior frontal fasciculus, inferior longitudinal fasciculus, and optic radiation.

A 62-year-old, right-handed male patient presented to the emergency department of the University Hospital of the Ludwig-Maximilians University, Munich, Germany, with signs of confusion, mild left hemiparesis, and peri-personal visuospatial neglect of the left hemifield. The confusion and hemiparesis were transient, lasting only a few hours, whereas the hemispatial neglect syndrome persisted. The patient completed paper–pencil neglect tests (the Bells test, Albert’s test), which disclosed pathologic scores consistent with spatial neglect [for detailed description of the tests, see in Ref. (15)]. The patient omitted all targets on the left side in the Albert’s test and all but three objects on the left side in the Bells test. He also exhibited a slowing of saccades toward the left hemifield and reduced optokinetic nystagmus to the left. A detailed neuro-orthoptic examination revealed no further deficits. Magnetic resonance imaging (MRI) showed an infarction in the parietal part of the right MCA territory which affected the IPL with the angular gyrus bordering and partly including the intraparietal sulcus (IPS), V5, and the temporo-occipital junction, but sparing the posterior insula, parietal opercular cortex, and posterior parietal cortex (PPC) (Figure 1).

### MR Imaging

Resting-state functional connectivity MRI was recorded before and after using caloric irrigation of the left (contralateral) ear with cold water (20°C) for 30 s to effect vestibular stimulation (16) on day 4 after stroke. To validate the effect of stimulation on behavior, neglect tests were carried out before the first imaging run (Albert’s test, the Bells test), immediately after stimulation (Albert’s test), and after the second imaging run [Albert’s test; for scoring see in Ref. (15)]. The same protocol was applied 6 months poststroke when the patient and his relatives reported normal spatial function. Normalization of neglect was confirmed on clinical examination and paper- and pencil-neglect tests (Albert’s test, the Bells test). The neuro-otological examination was also unremarkable.

Seed regions identified to be core regions of the central vestibular network in humans, e.g., the parietal opercular region (OP2) in the right and left hemispheres [Montreal Neurological Institute (MNI) coordinates [*x, y, z*: 40, −22, 16], [−48, −26, 18]] were chosen for fc analysis (8). Additionally, analyses were carried out for a region in the PPC [32, −48, 52] as this area was identified in the meta-analysis which revealed that saccadic eye movements are functionally connected with the vestibular cortical areas (8). Further, the following visual cortical areas were selected using seeds in V1, V2, the human occipital cortex 3d (hOC3d), and hOC5d (V5 only left), all of which were located outside the infarct area (17–19).

Images were acquired using a T2*-weighted echo planar imaging sequence (voxel size 3.5 mm × 3.5 mm × 3.5 mm, TE 30 ms, TR 1,900 ms, 36 slices, 190 volumes per run) on a GE 3 T scanner (GE Signa Excite HD, Milwaukee, WI, USA), equipped with an 8-channel head coil. Analysis was done using SPM12 (http://www.fil.ion.ucl.ac.uk/spm). Preprocessing included realignment, co-registration to the structural image, unified segmentation and normalization, followed by smoothing using an 8-mm Gaussian kernel.

The seed region’s time course was extracted at the voxel location in MNI space and temporally normalized (mean subtraction and scaling with standard deviation). All data from scanning before and after stimulation of the four imaging runs (acute phase pre/post stimulation; 6 months after stroke pre/post stimulation) were concatenated for analysis. Each seed region time course before and after intervention of the first and second measurement as well as the motion parameters and time courses of voxels within cerebrospinal fluid were used as regressors for the respective parts of the concatenated data in a fixed effects model in statistical parametric mapping (SPM) toolbox using first-level statistics, i.e., within subject. These include the sessions of acute pre, post and compensated pre, post as individual factors (one-way ANOVA). This allowed us to evaluate the differences in resting-state correlations with the seed region, before and after the intervention *via F*-tests and to evaluate differences between the measurements in the acute phase and 6 months later. The significance level was set to *p* < 0.05, the results were corrected for family-wise error (FWE), and clusters larger than 26 voxels were considered in the analysis. Selected significant clusters from this analysis and the original seed regions were used to compute intrinsic fc (correlation) between those regions in each of the four imaging runs. All Pearson correlation coefficients were Fisher *z*-transformed, the correlation threshold was set to 0.3, significance level *p* < 0.05, corrected for FWE.

### Standard Protocol Approvals, Registrations, and Patient Consent

The study was performed in accordance with the 1964 Declaration of Helsinki (last applicable revision 2008, Fortalzea) and was approved by the institutional review board of the University of Munich. The patient gave his written informed consent to participate in the study and for the publication of this case report.

## Results

In the acute phase the patient showed visuospatial hemineglect to the left on the behavioral level (see above) with neglect test scores that significantly improved in Albert’s test (no omission) after caloric irrigation.

### fc Analyses in the Acute Stage for Different Seed Regions

#### OP2 R

**Figure 2:**
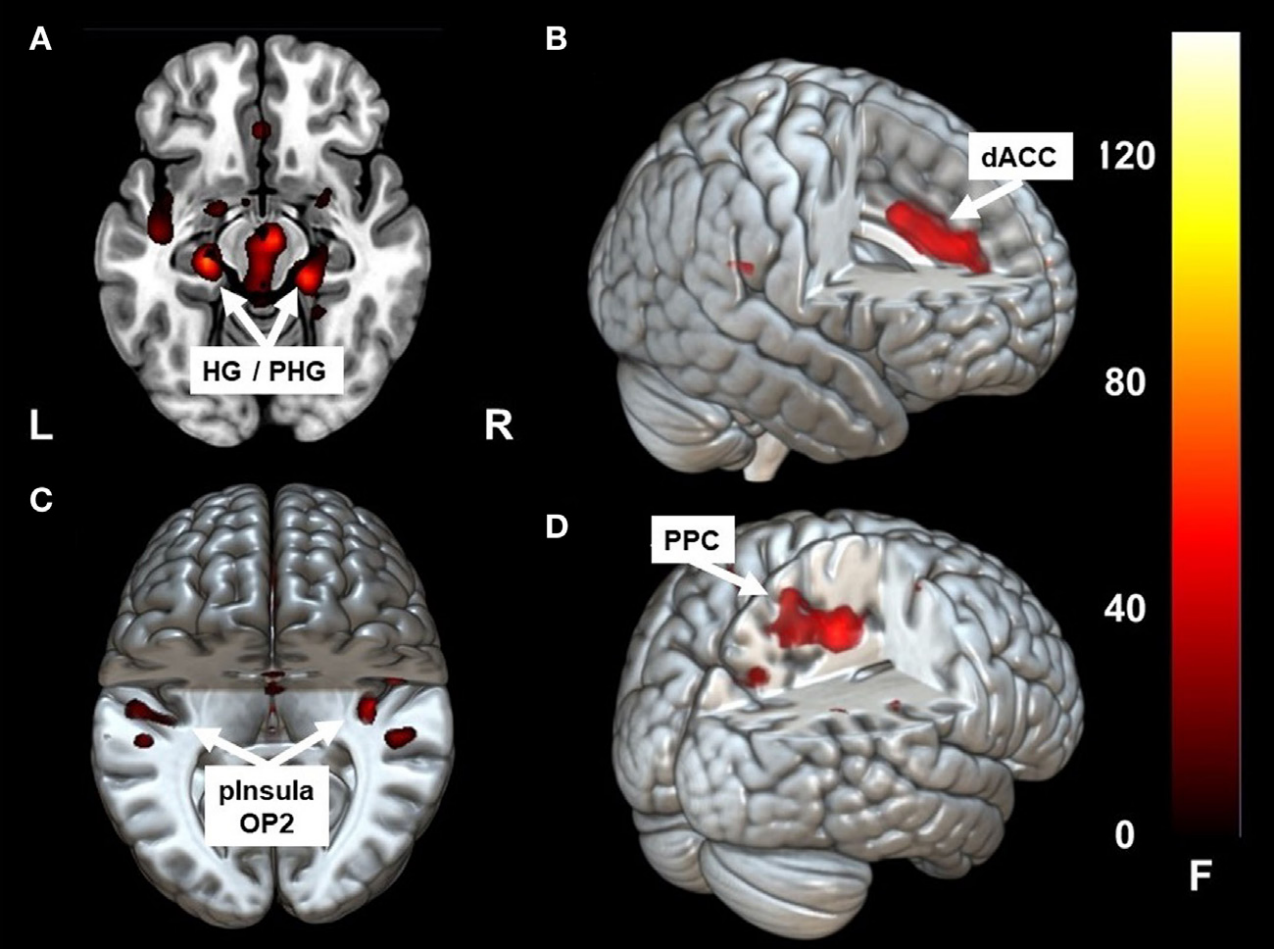
Clusters showing significant correlation with parietal operculum 2 (OP2) R. In the acute phase following caloric stimulation, a significant increase in correlation with OP2 R was found with **(A)** right and left hippocampus/para-hippocampus (HG/PHG) **(B)** dACC **(C)** left and right parieto-insular vestibular cortex. Panel **(D)** shows significant correlation with PPC after functional recovery (6 months at rest) compared to the acute phase at rest. Coordinates in Montreal Neurological Institute space are given in the text. OP2 parietal opercular cortex 2, dACC, dorsal anterior cingulate cortex; PPC, posterior parietal cortex. Cluster overlay on a template brain in MRICRO GL for visualization (http://www.mccauslandcenter.sc.edu/mricrogl/home).

**Figure 3:**
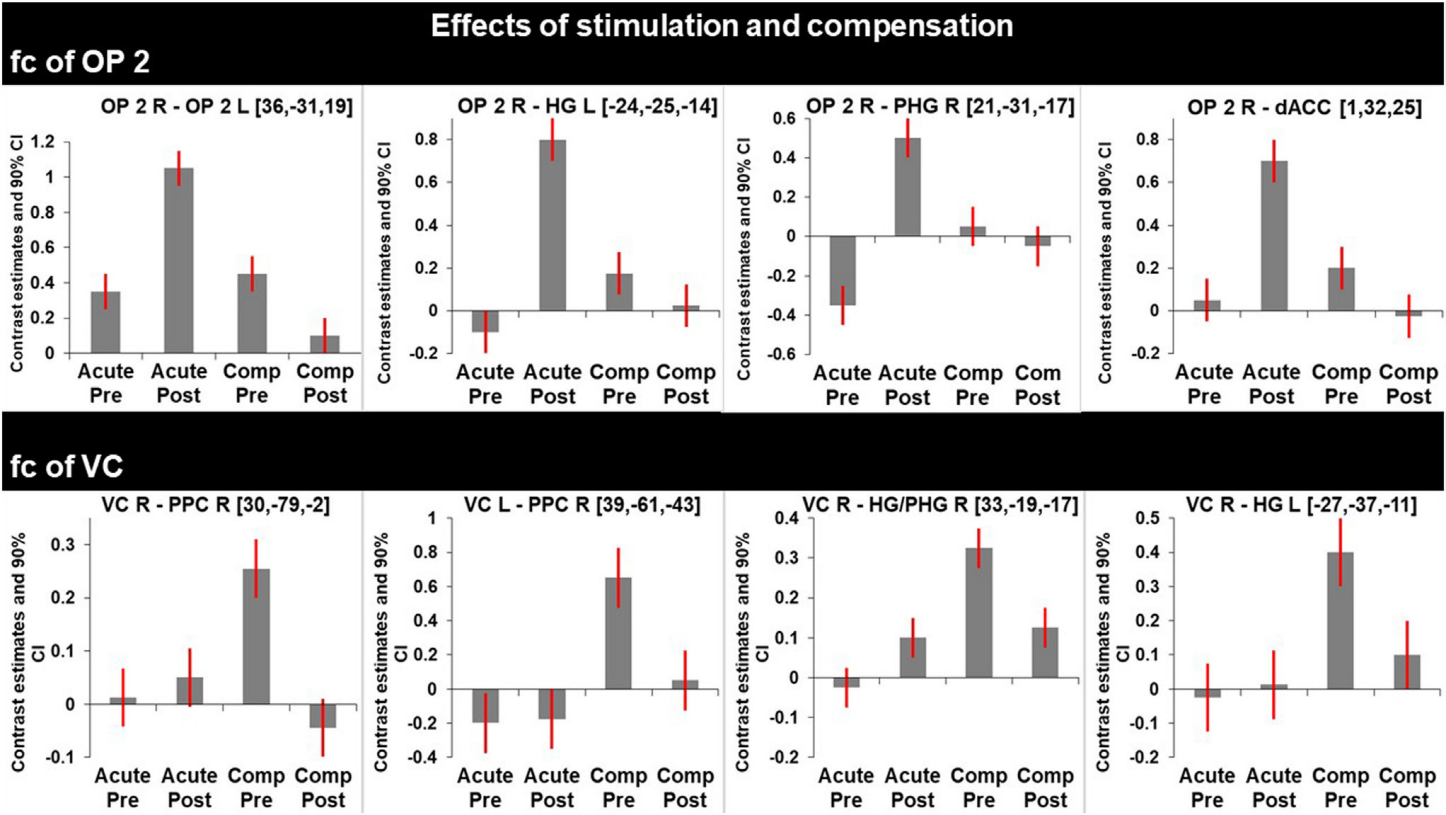
Graphs represent *F*-test effects of interest of regions with significant changes in functional connectivity (fc). Compared are the fc in the acute phase at rest (before) and shortly after caloric vestibular stimulation and in the chronic phase at rest and shortly after caloric vestibular stimulation. Note that there is increased fc within the bilateral multisensory vestibular network in the acute phase after stimulation while visual and multisensory integration centers for spatial orientation and navigation are disconnected in the acute phase and that fc between these regions is increased in the compensated phase at rest.

**Figure 4:**
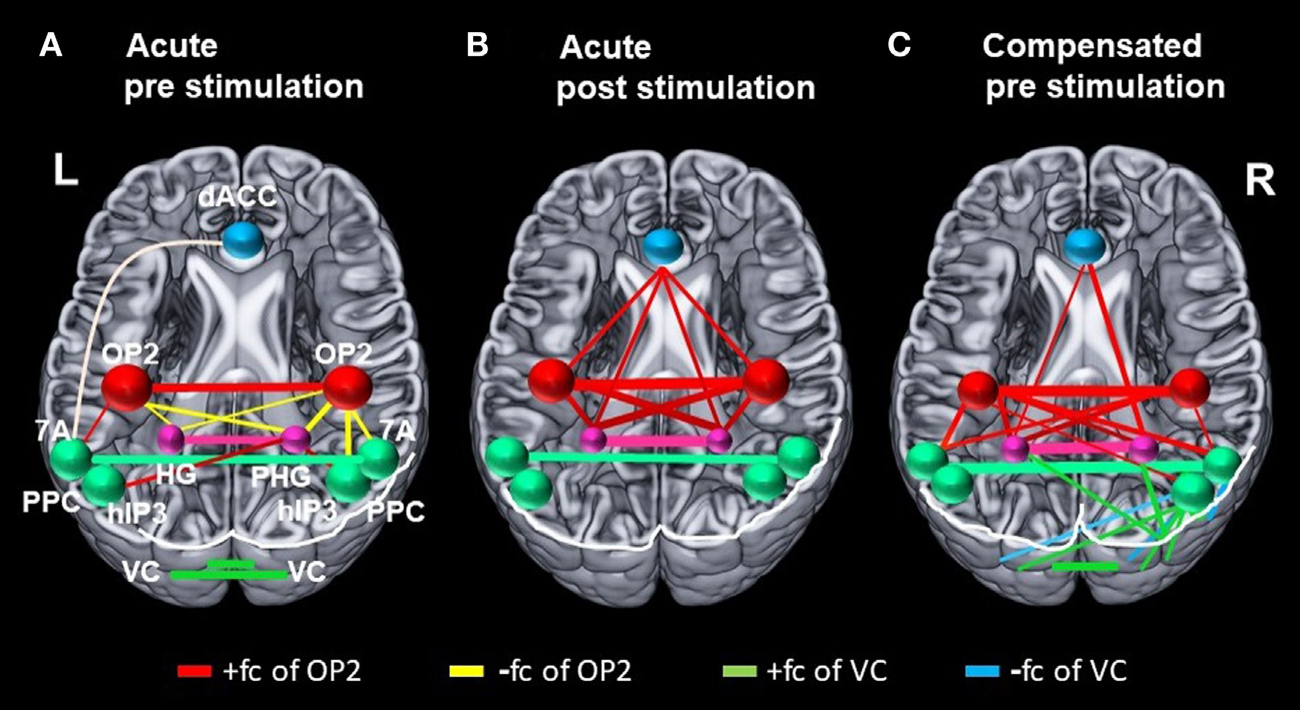
Changes in correlation (fc) of vestibular, visual, and multisensory areas. Schematic drawings of intrinsic functional connectivity (fc) changes over four imaging runs of selected seeds in the acute phase of the infarction and 6 months later after complete recovery from neglect with focus on the visual and vestibular system. Interhemispheric connectivity of the homologous regions posterior parietal cortex (PPC) (turquoise) and HG/PHG (pink) is shown in the color of the respective sphere. Bar diameter indicates strength of correlation. **(A)** anticorrelation between OP2 and posterior parietal cortex (PPC) as well as HG/PHG on both sides and strong interhemispheric connectivity of visual cortex (VC). **(B)** Increased interhemispheric functional connectivity (fc) of OP2 and with multisensory vestibular areas in dorsal anterior cingulate cortex (dACC) and bilateral HG/PHG while fc of VC is reduced. **(C)** Interhemispheric fc of OP2 and with multisensory vestibular areas in HG/PHG and of right OP2 with PPC as well as increased fc between VC with PPC and VC with HG/PHG.

Caloric irrigation during the acute phase led to increased fc in a network that included the parieto-temporo-insular region in both hemispheres, including on the left side the posterior insula, the parietal opercular cortex extending to the superior [superior temporal gyrus (STG)], and the middle temporal gyrus [−36, −31, 19]. The fc within the affected right hemisphere was relatively smaller and more restricted to the posterior insula, but it also reached the parietal opercular cortex and middle temporal gyrus [36, −31, 19]. Increased fc was also found to the dorsal anterior cingulum (dACC) [1, 32, 35] and to the ventral, mesial temporal regions along the parahippocampal gyrus and the hippocampus bilaterally [21, −31, −17], [−24, −25, −15] (Figures 2A–C; Figures 3 and 4A,B). Further, a cluster in area V2 [24, −49, −8] also showed increased connectivity to OP2 after stimulation during the acute phase.

#### OP2 L

A similar pattern was found when using the left OP2 as a seed region. Differences included extension of activation to the right STG and an additional increase of fc in the left mid-insula [−36, 8, −2] (Figure S1A in Supplementary Material).

#### Visual Cortex

After caloric irrigation a decrease of interhemispheric fc was observed in the acute phase between homologous regions in the VC areas (Figures 3 and 4A,B).

#### V5 L

Before and after caloric irrigation there was no fc to OP2 bilaterally, whereas there was increased fc to the left STG after stimulation (Figure S1B in Supplementary Material). Before but not after stimulation fc was increased to the left hippocampus. fc was also found to be increased to the PPC [to the left 7A before and after caloric stimulation, to the right human intraparietal sulcus 3 (hlP3) before stimulation]. fc to the VC was found bilaterally before stimulation and with a preference to the left VC after caloric stimulation.

### fc Analyses in the Chronic Stage

Similar to the caloric effects on fc during the acute stage, the connectivity pattern of the vestibular areas (OP2, HG/PHG, dACC) in the recovered stage without stimulation was enhanced compared to the initial resting state. Furthermore, without stimulation fc was increased between the cortical vestibular area *OP2 R* and the PPC on both sides [centering around areas 7A, 5M including areas 5ci, 7M and to a lesser extent the anterior IPS (hIP3) Figures 2D and 4C], as well as to visual areas V2 and V3. An additional cluster was found in the left middle frontal gyrus (not shown).

Caloric irrigation during the recovered stage 6 months later led to relatively decreased fc in these visual as well as vestibular areas, even those that had shown increased fc in the acute phase after caloric irrigation (Figure 3).

*Visual cortex (hOC3d) R* showed increased fc to the right hippocampus [33, −19, −17], within the VC, and the superior and medial frontal gyri [27, 14, 49] on the right (Figure S2A in Supplementary Material). Increased fc was also found for *V1/V2 R* to both IPL (not shown), as well as PPC R (hIP3) to V4 on the right and PPC on the left side (Figure S2B in Supplementary Material). PPC showed increased interhemispheric fc in the compensated phase at rest (Figure S3 in Supplementary Material).

Before stimulation *V5 L (hOC5d)* showed increased fc to the left OP2 and the hippocampus, VC, and PPC (7A L, hlP3 R) bilaterally. After caloric stimulation there was no fc to either OP2 or PPC, but persistent fc to both VC and both hippocampi (L > R).

## Discussion

Up to now there have been only a few fc studies which focused on inter- and intrahemispheric resting-state networks in neglect patients. In one resting-state fMRI experiment a reduced activation in distinct visual (lateral occipital cortex) and parietal cortex areas of the right hemisphere was seen as specific correlates of neglect, suggesting a disturbed modulation of visual processing by the parietal cortex (20). Furthermore, in this study an asymmetry of parietal cortex activation (relative hyperactivation of the left) was described in neglect as well as in stroke control patients without neglect (20). Recent fMRI studies have shown decreased interhemispheric connectivity of homologous regions in the acute stage of neglect across multiple resting-state networks, intrahemispheric decreases of anticorrelation between default mode and ventral attention/motor networks. Increased interhemispheric fc after recovery as well as a relative increase in anticorrelation of the aforementioned networks after compensation was also found (21, 22).

Our study focusing on the vestibular and visual networks detected changes in fc which were elicited by caloric vestibular stimulation in the acute stage of neglect. These changes were largely similar to changes in fc found in the resting state after recovery. In the acute stage, caloric stimulation particularly strengthened the transhemispheric connections within the vestibular network (insula, OP2, GTS, hippocampus, ACC), but weakened the transhemispheric connections between visual cortical areas. After functional recovery from neglect the fc at rest was similar to that of the vestibular network, but it differed in the intra- and transhemispheric visual-vestibular, and visuo-visual connections. In the following, we discuss similarities and differences between the effects of the transient changes elicited by caloric irrigation in the acute stage and the stable reorganization after recovery.

## Mechanisms of Improvement of Spatial Orientation Following Caloric Irrigation in the Acute Phase

The effect of caloric stimulation on fc of OP2 was associated with an improved performance in Albert’s test which lasted about 20 min. This is in line with reports of caloric vestibular stimulation in neglect patients (23). The pattern of increased fc corresponds to that of activations during stimulation in healthy volunteers reported in positron emission tomography/fMRI studies (7, 8). It is noteworthy that there was a time gap of 5 min between caloric stimulation and start of the imaging run due to the performance of the neglect test. This (prolonged) effect of caloric stimulation on changes in the resting-state connectivity has not been shown before.

While rostral and mesial temporal regions in both hemispheres, which are involved in spatial perception and navigation (STG, MTG, hippocampus), showed stronger connectivity to OP2 after caloric stimulation, such an effect was not observed for the parietal regions (PPC), which are involved in spatial orientation to the contralateral left hemispace (Figures 2A,C and 4B). A direct influence of vestibular signals on the left PPC in the sense of sensory substitution or multisensory integration *via* enhanced visual input in the acute phase seems, therefore, unlikely. In contrast, the lack of interaction of the vestibular cortex and the PPC bilaterally implies that this transient improvement relies on a different mechanism, possibly an increase of the sensorial weight of the vestibular input to multisensory integration for spatial attention. The bilaterally increased fc between the vestibular cortex, the (para-)hippocampus, and the ACC may provide the structural basis for such a mechanism.

The maps for spatial orientation and navigation are created in the mesial temporal lobe. In brief, this is achieved by integrating landmark-based visual cues of the external environment with information about the position and movement of eyes, head, and body; this information is mediated by specialized cell populations in the hippocampus and entorhinal cortex [for detailed reviews see Ref. (24, 25)]. Vestibular responses in these regions have been shown in rodents and humans (13, 26). Indeed, neglect has also been reported in patients with parahippocampal lesions (27). Along with connections *via* the PPC (which was functionally disconnected), a pattern involving medial prefrontal cortex and dorsal anterior cingulum has been described (28). Our patient also showed such a pattern of increased fc, suggesting that the fc possibly takes this route between these areas.

Furthermore, while the interhemispheric connectivity between vestibular areas was increased, we found that the interhemispheric connectivity between homologous regions of the VC was reduced after caloric irrigation in the acute phase. This might have led to a reduced interhemispheric inhibition of the right VC after vestibular stimulation. It had been hypothesized earlier in a model approach that a lesion-induced neglect reduces the interhemispheric crosstalk (29). The reduced interhemispheric connectivity of VC regions is in line with the data of recent fc studies (21, 22). However, it reflects only one component of the complex vestibular–visual interactions in which the increase of the interhemispheric connectivity between vestibular areas plays a prominent role.

## fc Changes in the Recovered Stage in Vestibular and VC Areas

Like the fc pattern in the acute phase after caloric stimulation, the interhemispheric connectivity of the vestibular areas (OP2, hippocampus, dACC) was enhanced in the resting state after recovery from neglect 6 months later. Moreover, in contrast to the connectivity pattern in the acute phase, recovery was accompanied by increased fc of OP2 to the PPC (7A) on both sides and to visual areas in the right hemisphere. Connectivity between PPC (hIP3) and VC areas was also increased between both hemispheres. Additionally, increased fc was found between the right visual area hOC3d and both hippocampi, possibly representing substitutional processes of visual–vestibular interactions.

This implies that the repair mechanisms—as seen in the resting state without stimulation—show within-months similarities to as well as differences from the short-term improvement of neglect following caloric irrigation. The observed increase in fc of the vestibular cortex OP2 to the PPC indicates that multisensory signal integration was intensified over 6 months (Figure 3). The finding that the fc to multisensory vestibular areas is decreased following caloric stimulation in the compensated phase and increased between visual area hOC3d and the hippocampi might be due to a retuning of multisensory neurons to respond preferably to visual stimuli (i.e., substitution). Compared with other sensory systems, a specific feature of the cortical vestibular network is the multimodal nature of neural responses (30–32). While the increased tuning of the multisensory brain areas to visual cues led to compensation of the deficit at rest, vestibular stimulation created a sensory mismatch in the compensated phase. Usually a mismatch is suppressed by inhibitory visual–vestibular interactions (33), but in this case the higher preference for visual stimuli in the compensated phase might have led to an alteration of visual–vestibular interaction.

The presence of edema in the acute phase as well as structural reorganization of the damaged tissue in the chronic phase might also have contributed to the increase in fc between visual and parietal cortex. However, the increase of fc in vestibular cortical areas following vestibular stimulation in the acute phase in comparison to the relative decrease following stimulation after recovery suggest functional rather than structural reorganization.

## Conclusion

To conclude, caloric irrigation led to changes in fc that imply the existence of a beneficial (short-term) mechanism that relies on an enhanced vestibular input to a multisensory integration process. The process is mediated by increased fc of the vestibular cortex to mesial temporal lobe areas involved in visual–vestibular signal integration for spatial attention, perception, memory, and navigation. This led to reduced interhemispheric connectivity in both VC. Functional recovery of the hemineglect 6 months later was associated with (i) fc increases of the ipsilateral vestibular cortex to VC areas of the right hemisphere, (ii) enhanced interhemispheric PPC connectivity, and (iii) fc increases between VC areas and PPC within the ipsilateral hemisphere. These pattern changes might be based on the tuning and enhancing of the sensorial weight of vision in multisensory neuronal assemblies in the parietal and insular cortex.

The fc data cannot answer the question of whether these connections are excitatory or inhibitory. It has been shown for the visual system that most transcallosal networks are inhibitory (34, 35). Therefore, models were proposed which explained the deficits of neglect by interhemispheric inhibition (29, 34, 35). However, excitatory transcallosal connections have been described for MT/V5 after motion stimulation of patients with complete homonymous hemianopia (36) and in an fMRI study using coherent visual motion stimulation of the right or left visual hemifield in healthy volunteers (37). To date, transcallosal vestibular connections have been demonstrated in humans by tracking fibers with diffusion tensor imaging (38), but it is not known whether the fc is inhibitory, excitatory, or both.

A further limitation of our study is that the changes in fc have been observed in a single patient whose spatial hemineglect was perceptional and deficient in peri-personal space and space perception/navigation. It remains an open question whether the observed fc changes associated with vestibular stimulation and the clinical course of our neglect patient are typical for this syndrome or whether other patients and other neglect subtypes show different neuronal reorganizations associated with functional recovery.

## Ethics Statement

The study was performed in accordance with the 1964 Declaration of Helsinki (last applicable revision 2008, Fortalzea) and was approved by the institutional review board of the University of Munich. The patient gave his written informed consent to participate in the study and to publication of this case report.

## Author Contributions

JC: drafting/revising the manuscript, study concept and design, acquisition of data, and analysis and interpretation of data. RB: drafting/revising the manuscript and analysis and interpretation of data. ME: drafting/revising the manuscript and acquisition, analysis and interpretation of data. TB: critical revision of the manuscript for important intellectual content, study supervision, and obtaining funding. MD: drafting/revising the manuscript, study concept and design, analysis and interpretation of data, study supervision, obtaining funding.

## Conflict of Interest Statement

The authors declare that the research was conducted in the absence of any commercial or financial relationships that could be construed as a potential conflict of interest.

## Supplementary Material

Figure S1 Clusters showing significant correlation with parietal operculum 2 (OP2) L and hOC5d L in the acute phase. **(A)** After caloric vestibular stimulation in the acute phase, left OP2 shows significant functional connectivity (fc) with right OP2, dorsal anterior cingulate cortex (dACC), left and right (para-) hippocampal gyrus, and left superior temporal gyrus (STG). **(B)** hOC5d L shows significant fc with left STG following caloric stimulation in the acute phase. Statistical parametric mapping maps overlay on a template brain in MRICro GL for visualization (http://www.mccauslandcenter.sc.edu/mricrogl/home).

Figure S2 Clusters showing significant correlation with visual cortex (VC) R in the compensated phase (functional recovery). **(A)** Functional connectivity (fc) of human occipital cortex 3 dorsal R is increased in the compensated phase at rest (functional recovery) compared to the acute phase at rest with clusters in the right hippocampus and right middle/superior frontal gyrus. **(B)** fc of V2 R in the compensated phase at rest (functional recovery) shows increased fc with VC and posterior parietal cortex (PPC). Statistical parametric mapping maps overlay on a template brain in MRICro GL for visualization (http://www.mccauslandcenter.sc.edu/mricrogl/home).

Figure S3 Clusters showing significant correlation with posterior parietal cortex (PPC) R in the compensated phase (functional recovery). Increased interhemispheric connectivity between both PPC in the compensated phase at rest (functional recovery) compared to the acute phase without stimulation. Statistical parametric mapping maps overlay on a template brain in MRICro GL for visualization.

Figure S4 Correlation of seeds following caloric stimulation in the compensated phase. Correlation of seed regions following caloric stimulation in the compensated phase. Assignment of lines: red: positive correlation (fc) of parietal operculum 2 (OP2) (red dots); dark blue: negative correlation with OP2; green: positive correlation of visual cortex (VC) with VC; light blue: negative correlation of posterior parietal cortex (PPC) with VC. Bar diameter indicates strength of correlation. Interhemispheric connectivity of the homologous regions PPC (turquoise) and HG/PHG (pink) is shown in the color of the respective sphere.

## Abbreviations

CSF, cerebrospinal fluid; dACC, dorsal anterior cingulate cortex; EPI, echo planar imaging; fc, functional connectivity; FWE, family-wise error; hOC3d, human occipital cortex 3 dorsal; hlP3, human intraparietal sulcus 3; IPL, inferior parietal lobule; IPS, intraparietal sulcus; MCA, middle cerebral artery; MFG, middle frontal gyrus; MNI, Montreal Neurological Institute; MRI, magnetic resonance imaging; OP2, parietal operculum 2; PET, positron emission tomography; PPC, posterior parietal cortex; SPM, statistical parametric mapping; STG, superior temporal gyrus.
