# Supplementary figures and images for: Recovery from Spatial Neglect with Intra- and Transhemispheric Functional Connectivity Changes in Vestibular and Visual Cortex Areas—A Case Study
Source: Front Neurol. 2018 Mar 2;9:112. doi: 10.3389/fneur.2018.00112 (PMC5840167; doi:10.3389/fneur.2018.00112)

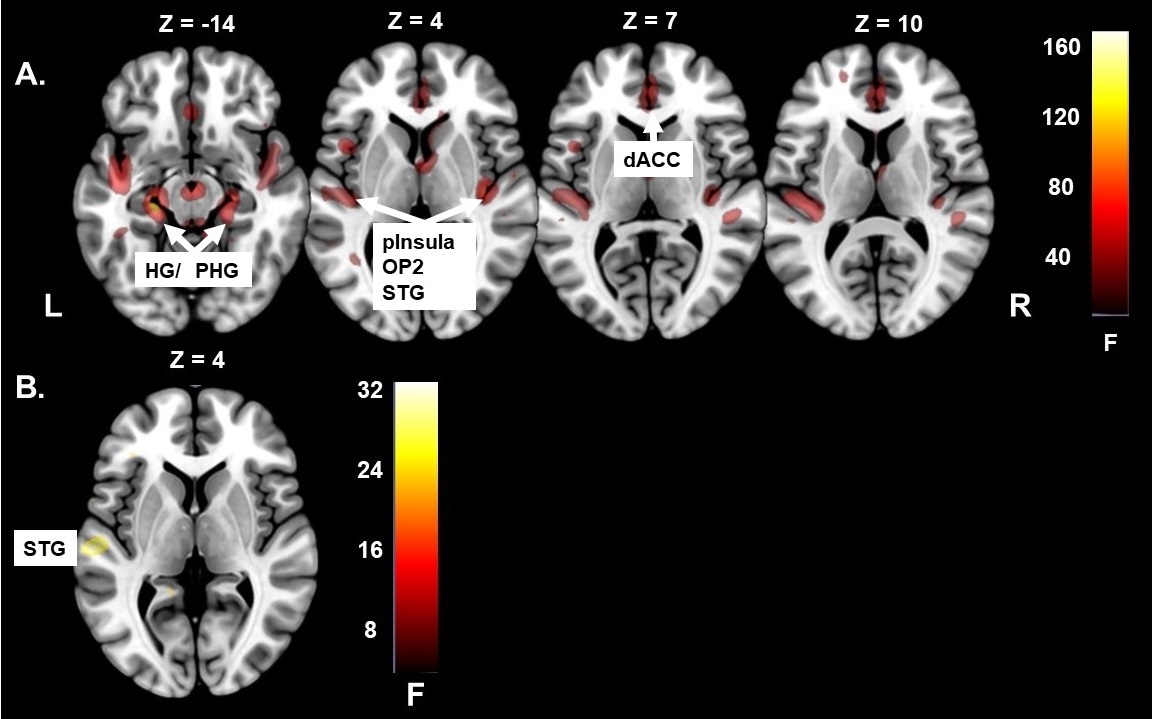

Supplement: Figure S1 — Clusters showing significant correlation with parietal operculum 2 (OP2) L and hOC5d L in the acute phase. (A) After caloric vestibular stimulation in the acute phase, left OP2 shows significant functional connectivity (fc) with right OP2, dorsal anterior cingulate cortex (dACC), left and right (para-) hippocampal gyrus, and left superior temporal gyrus (STG). (B) hOC5d L shows significant fc with left STG following caloric stimulation in the acute phase. Statistical parametric mapping maps overlay on a template brain in MRICro GL for visualization (http://www.mccauslandcenter.sc.edu/mricrogl/home). [file image_1.jpeg]

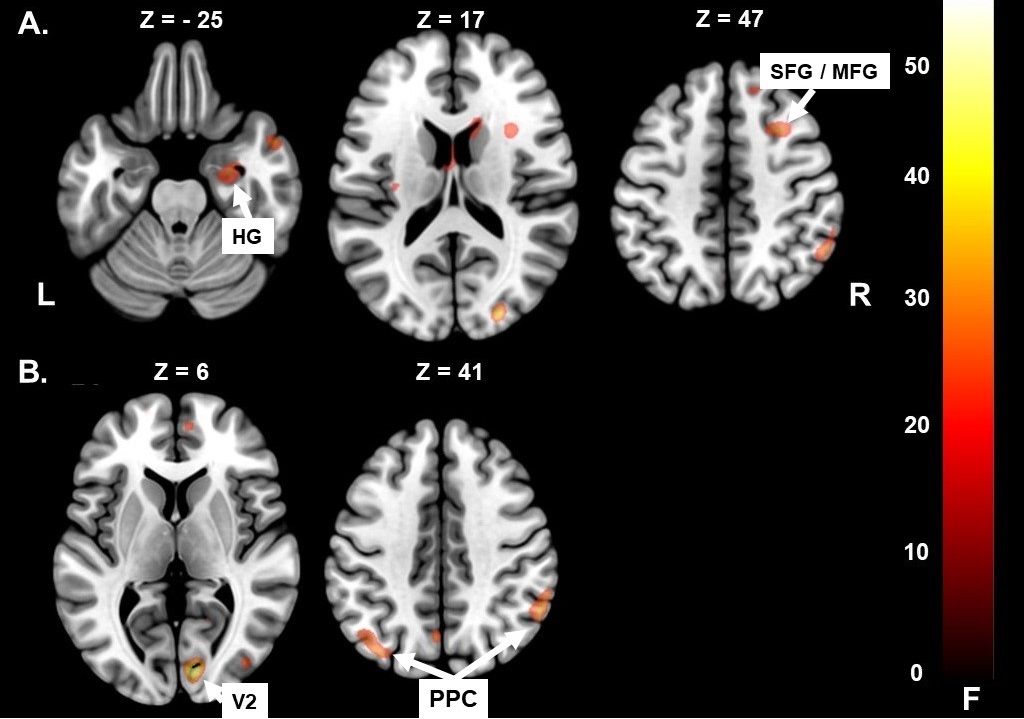

Supplement: Figure S2 — Clusters showing significant correlation with visual cortex (VC) R in the compensated phase (functional recovery). (A) Functional connectivity (fc) of human occipital cortex 3 dorsal R is increased in the compensated phase at rest (functional recovery) compared to the acute phase at rest with clusters in the right hippocampus and right middle/superior frontal gyrus. (B) fc of V2 R in the compensated phase at rest (functional recovery) shows increased fc with VC and posterior parietal cortex (PPC). Statistical parametric mapping maps overlay on a template brain in MRICro GL for visualization (http://www.mccauslandcenter.sc.edu/mricrogl/home). [file image_2.jpeg]

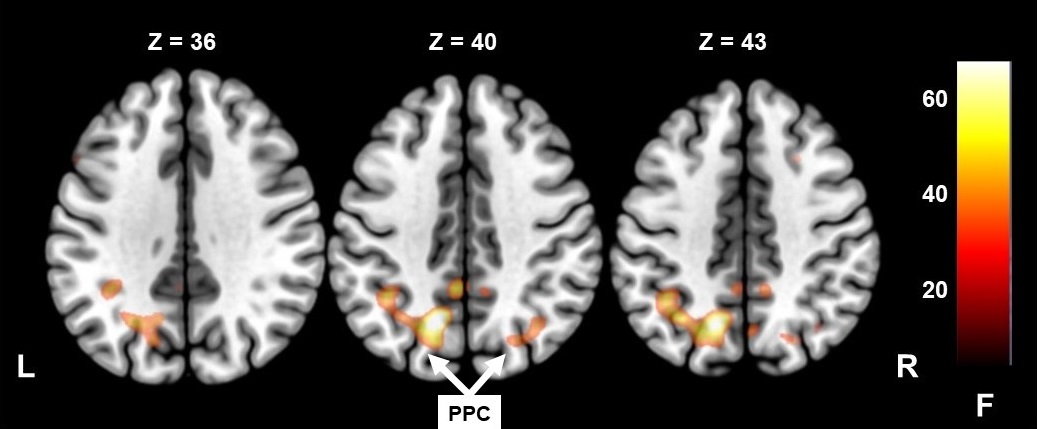

Supplement: Figure S3 — Clusters showing significant correlation with posterior parietal cortex (PPC) R in the compensated phase (functional recovery). Increased interhemispheric connectivity between both PPC in the compensated phase at rest (functional recovery) compared to the acute phase without stimulation. Statistical parametric mapping maps overlay on a template brain in MRICro GL for visualization. [file image_3.jpeg]

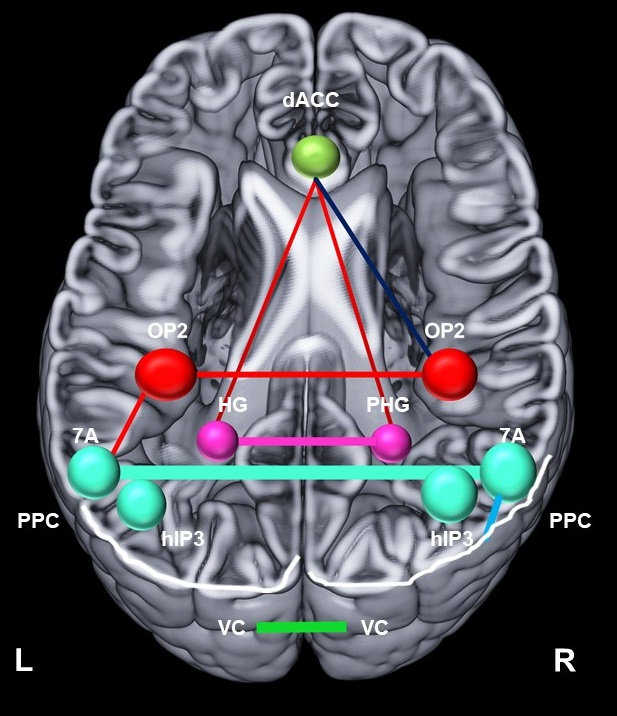

Supplement: Figure S4 — Correlation of seeds following caloric stimulation in the compensated phase. Correlation of seed regions following caloric stimulation in the compensated phase. Assignment of lines: red: positive correlation (fc) of parietal operculum 2 (OP2) (red dots); dark blue: negative correlation with OP2; green: positive correlation of visual cortex (VC) with VC; light blue: negative correlation of posterior parietal cortex (PPC) with VC. Bar diameter indicates strength of correlation. Interhemispheric connectivity of the homologous regions PPC (turquoise) and HG/PHG (pink) is shown in the color of the respective sphere. [file image_4.jpeg]
